# Velocity-Constraint Kalman Filtering for Enhanced Bubble Tracking in Motion-Compensated Ultrasound Localization Microscopy

**DOI:** 10.34133/research.0725

**Published:** 2025-06-04

**Authors:** Yifei Zhu, Lingyin Jiang, Qi Zhang, Jun Yin, Bingze Du, Guofeng Zhang, Haijun Zhang, Bo Ding, Han Lin, Honghui Xue, Xiasheng Guo, Xiao-Yang Zhang, Jing-Ning Zhu, Dong Zhang, Juan Tu, Ning Gu

**Affiliations:** ^1^ Key Laboratory of Modern Acoustics, Department of Physics, Nanjing University, Nanjing 210093, China.; ^2^ School of Computer and Electronic Information, Nanjing Normal University, Nanjing 210023, China.; ^3^ State Key Laboratory of Pharmaceutical Biotechnology, National Resource Center for Mutant Mice, Department of Anesthesiology, Nanjing Drum Tower Hospital, Institute for Brain Sciences, and Department of Physiology, School of Life Sciences, Nanjing University, Nanjing 210023, China.; ^4^ Shanghai Tenth People’s Hospital, School of Medicine, Tongji University, Shanghai 200092, China.; ^5^ National United Engineering Laboratory for Biomedical Material Modification, Branden Industrial Park, Dezhou 251100, Shandong, China.; ^6^ Zhuhai Ecare Electronics Science & Technology Co., Ltd., Zhuhai 519041, China.; ^7^ School of Engineering Audit, Nanjing Audit University, Nanjing 211815, China.; ^8^ Nanjing Key Laboratory for Cardiovascular Information and Health Engineering Medicine, Institute of Clinical Medicine, Cardiovascular Medical Center, Nanjing Drum Tower Hospital, Medical School, Nanjing University, Nanjing 210093, China.

## Abstract

Ultrasound localization microscopy (ULM) is a novel imaging technique that overcomes the diffraction limit to achieve super-resolution imaging at the 10-μm scale. Despite its remarkable progress, challenges persist in enhancing the precision of microbubble tracking and fulfilling the requirements for high frame rates in practical circumstances, especially in moving organs. To address these issues, an enhanced ULM approach (shorten as vc-Kalman) integrating rapid motion compensation was developed to achieve excellent image quality. Unlike traditional methods relying on observed bubble positions, the proposed algorithm combined statistical information derived from historical data with Kalman-filter-predicted positions to enable more accurate bubble localization. Meanwhile, microbubble brightness in adjacent frames was incorporated as multidimensional feature to further improve the matching efficacy. Furthermore, velocity constraint was applied to minimize possible erroneous traces and enhance the contrast-to-noise ratio of ULM images, while ensuring the continuity of vascular reconstruction and the accuracy of the blood flow analysis to generate a reduced normalized root mean square error in velocity estimation, even at a relatively low frame rate of 146 Hz. More important, to effectively suppress the impact of physiological movements in moving organs like kidneys, this algorithm fulfilled subpixel displacement vector identification through parabolic fitting and expedited motion compensation via dynamic programming-based cross-correlation search. The results indicated that this advanced vc-Kalman method substantially boosted both the robustness and accuracy of ULM imaging, thereby opening more opportunities for clinical applications of super-resolution ULM technology.

## Introduction

Ultrasound has become one of the most widely used clinical diagnostic tools due to its unique advantages, such as noninvasiveness, nonionizing radiation, ease of use, and the ability to focus on deep tissues [1,2]. However, compared to imaging modalities like computed tomography and magnetic resonance imaging, the clinical applications of ultrasound imaging are normally limited by its relatively low resolution. Thus, researchers have attempted to improve the ultrasound image resolution by increasing the frequency and/or reducing the pulse length, although these methods might result in the reduction of acoustic wave penetration depths. Advanced imaging algorithms and postprocessing techniques have also been developed to offer better diagnostic performance [3,4], but are also constrained by the Rayleigh criterion, preventing them from overcoming the diffraction limit of sound waves.

Betzig et al. [5] firstly proposed an optical super-resolution imaging method that won the Nobel Prize in Chemistry in 2014. This technique surpasses the diffraction limit by localizing sparsely distributed fluorescent probes and accumulating their positions over time to generate super-resolution image [6]. By extending this groundbreaking concept to medical ultrasound areas, Couture et al. [7] proposed a novel imaging modality named ultrasound localization microscopy (ULM). The core of ULM’s super-resolution capability lies in precisely tracking microbubble agents in blood vessels across multiple frames, and subsequently achieving subwavelength spatial resolution in ultrasound images by accumulating microbubble trajectory data over time.

Ultrasound contrast microbubbles, with a size comparable to that of red blood cells, can reach areas where red blood cells flow. Due to their precisely tunable intravascular administration concentration, these bubbles can serve as effective tracking agents in ULM to provide satisfactory localization and tracking efficacy. Meanwhile, the strong acoustic scattering properties of these bubbles make them easier to track within the microvessels. Therefore, unlike traditional ultrasound imaging, ULM enables the capability to capture subtle dynamic changes in microvascular blood flow. Especially compared with traditional Doppler methods, ULM imaging can even improve its spatial resolution by several orders of magnitude [8], providing researchers with rich and accurate data that might promote significant progress in related fields [9]. For instance, Renaudin et al. [10] used ULM to generate dynamic microflow videos, enabling detailed observation of blood flow changes in the rat brain in response to whisker and visual stimulation, which suggested that ULM should be able to reveal previously unobservable micro-blood flow details and offer valuable insights for neuroscience research. In addition, the ULM method has been successfully utilized to study organs, such as the ear, kidneys, and tumors in animals [11–13], and it has shown promising results in human organs, including the brain, heart, thyroid, breast, and liver [14–16].

Despite significant advancements, the practical applications of ULM still face critical challenges, wherein the greatest difficulties come from enhancing the precision of microbubble matching and localization algorithms [17–22]. For example, early ULM algorithms addressed matching errors by limiting microbubble counts [20]. High bubble concentrations may lead to overlapping signals and increased interference, resulting in blurred and inconsistent microvascular network reconstruction. Appropriately limiting microbubble concentration helps minimize the likelihood of signal interference from multiple nearby microbubbles, thereby improving the accuracy of microbubble localization in ULM and reducing matching errors. However, the limited number of detectable microbubbles within a given accumulation time hinders the image quality enhancement of ULM, especially under low frame rate conditions, leading to discontinuous vascular imaging and unreliable flow estimations. Although the increase in the frame rate can improve microbubble tracking precision and matching accuracy, it also increases acoustic output, which shortens the bubble lifespan, limits imaging depth, and results in larger data volume, which certainly adds considerable data transmission and processing burdens. Therefore, later studies employed methods like Kalman filtering [18] to correct bubble tracking errors, while neural networks [19–21] have also shown potential in high-density microbubble matching. However, the complexity and computational demands of neural networks remain substantial, especially in complex flow environments.

Nevertheless, practical applications remain constrained by factors such as limited acquisition time (due to considerations like anesthesia and microbubble injection volume), complex flow patterns (e.g., tortuous or intersecting vessels), and the computational complexity of neural network algorithms. These challenges make it difficult to achieve rapid and precise microbubble localization, and accurate matching of large numbers of moving bubbles using current ULM algorithms, significantly limiting the clinical translation of ULM technology. More critically, in vivo imaging is inevitably affected by displacement and deformation caused by physiological activities (e.g., heartbeats, respiration, and muscle contractions). The development and application of robust motion compensation algorithms [23] are therefore essential to further improve the accuracy of cross-frame matching and image reconstruction.

To overcome the above obstacles, the present work proposed a novel matching algorithm that predicted subsequent positions of individual microbubbles more accurately by integrating both the historical and statistically predicted information of microbubble velocities, rather than relying solely on their position data in the previous frame. Then, the matching algorithm was processed between the predicted and actually observed positions for individual bubbles in subsequent frames. Additionally, it incorporated brightness information as a multidimensional feature to further improve the matching efficacy and took velocity constraints into account to enhance the quality of ULM images. Testing on rat brain images demonstrated that the present approach would significantly enhance the accuracy and robustness of microbubble localization and tracking with reduced computational burden, enabling more efficient matching and more accurate blood flow analysis to generate enhanced image resolution and reduced velocity estimation errors, even at even relatively low frame rates. More important, when applied to moving organs, such as the kidney, this algorithm even provided subpixel displacement vector identification through parabolic fitting and introduced optimized motion compensation via expedited normalized cross-correlation search to effectively suppress the impact of physiological movements like heartbeats, demonstrating satisfactory results and highlighting strong potential for broader clinical applications.

## Results

**Fig. 1.**
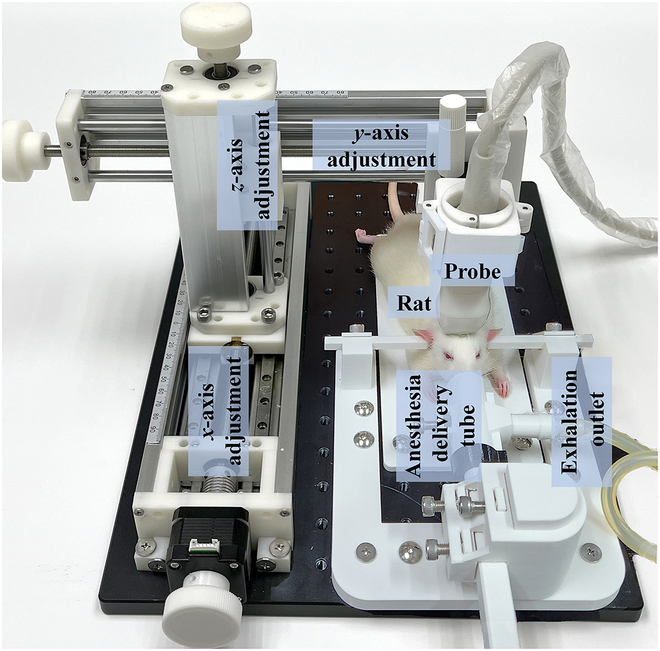
Illustration of the 3D scanning device for animal experiments.

As depicted in Fig. 1, a 3-dimensional (3D) scanning device was used to perform ULM imaging on the animals, which includes a triaxial movement platform with 10-μm fine-tuning capabilities on each axis. The ULM imaging was performed 1 min after the injection of ultrasound contrast agents to ensure stable microbubble distribution. The imaging module (Zhuhai Ecare, China) was utilized to continuously capture 100,000-frame radiofrequency (RF) data with an ultrafast frame rate up to 440 fps (which is 10 times higher than regular frame rate) and then transferred to PC for following processing to achieve super-resolution ULM images in rat’s brain or kidney.

**Fig. 2.**
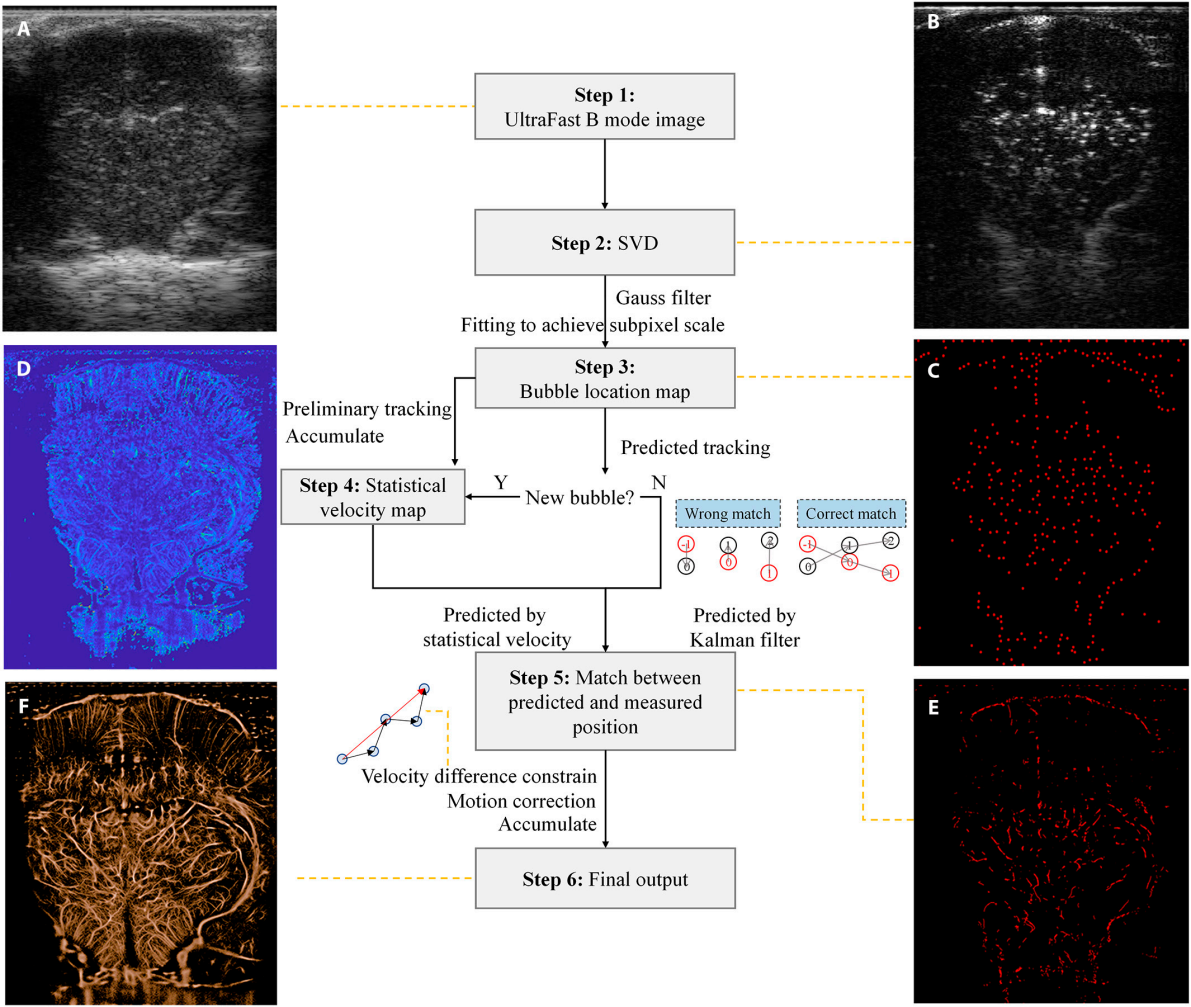
Flowchart of velocity-constrained Kalman filtering for enhanced bubble tracking in motion-compensated super-resolution ultrasound localization microscopy: (A) ultrafast B mode image; (B) image after SVD; (C) bubble localization map; (D) velocity map; (E) bubble trajectories construction after matching; (F) final output image.

Figure 2 demonstrates the step-by-step computational workflow for the proposed velocity-constrained Kalman filtering method (referred as vc-Kalman) with enhanced bubble tracking accuracy in motion-compensated super-resolution ULM. In brief, B-mode images with high temporal resolution are initially created from the raw data acquired in an ultrafast mode with a frame rate up to 440 fps (Fig. 2A). All these raw data contain both tissue echoes and microbubble signals. Then, singular value decomposition (SVD) is applied to filter out tissue clutter with slow spatial–temporal variations, thereby isolating microbubble signals (Fig. 2B). It should be noticed that, a noise threshold, empirically determined in advance from control measurements without microbubble injection, is used to refine the SVD signal separation, ensuring that only microbubble-originated signals above the noise threshold can be retained. In step 3, in order to achieve a high-precision microbubble localization map, Gaussian filtering is first applied to enhance the signal-to-noise ratio. Then, a local cosine function fitting is performed around individual bubbles to determine bubble centroid positions with subpixel resolution (Fig. 2C). Following this, a preliminary bubble tracking process is carried out to generate an initial velocity map by associating bubble positions across consecutive frames based on spatial proximity (Fig. 2D). However, this basic matching process may suffer from errors due to complex motion patterns and intermittent detection losses. To address matching errors and ambiguities, an extended Kalman filter framework is implemented in the following step, utilizing both Kalman prediction and historically statistical velocity information to track bubble trajectories and validate matching candidates probabilistically (Fig. 2E). Additionally, microbubble brightness consistency is employed as an auxiliary constraint to further improve the matching robustness. In the final stage, dynamic motion compensation is applied to correct for tissue movements (e.g., respiration and cardiac pulsations) based on estimated displacement fields. Trajectories are further refined by applying a velocity difference (VD) constraint to filter out inconsistent tracks. Eventually, the accumulated bubble trajectories are reconstructed into a super-resolution ULM vascular image with improved spatial and flow accuracy (Fig. 2F). Detailed information regarding the crucial innovative processing will be introduced in Materials and Methods.

### Enhanced bubble tracking effect induced by velocity-constrained Kalman filtering

**Fig. 3.**
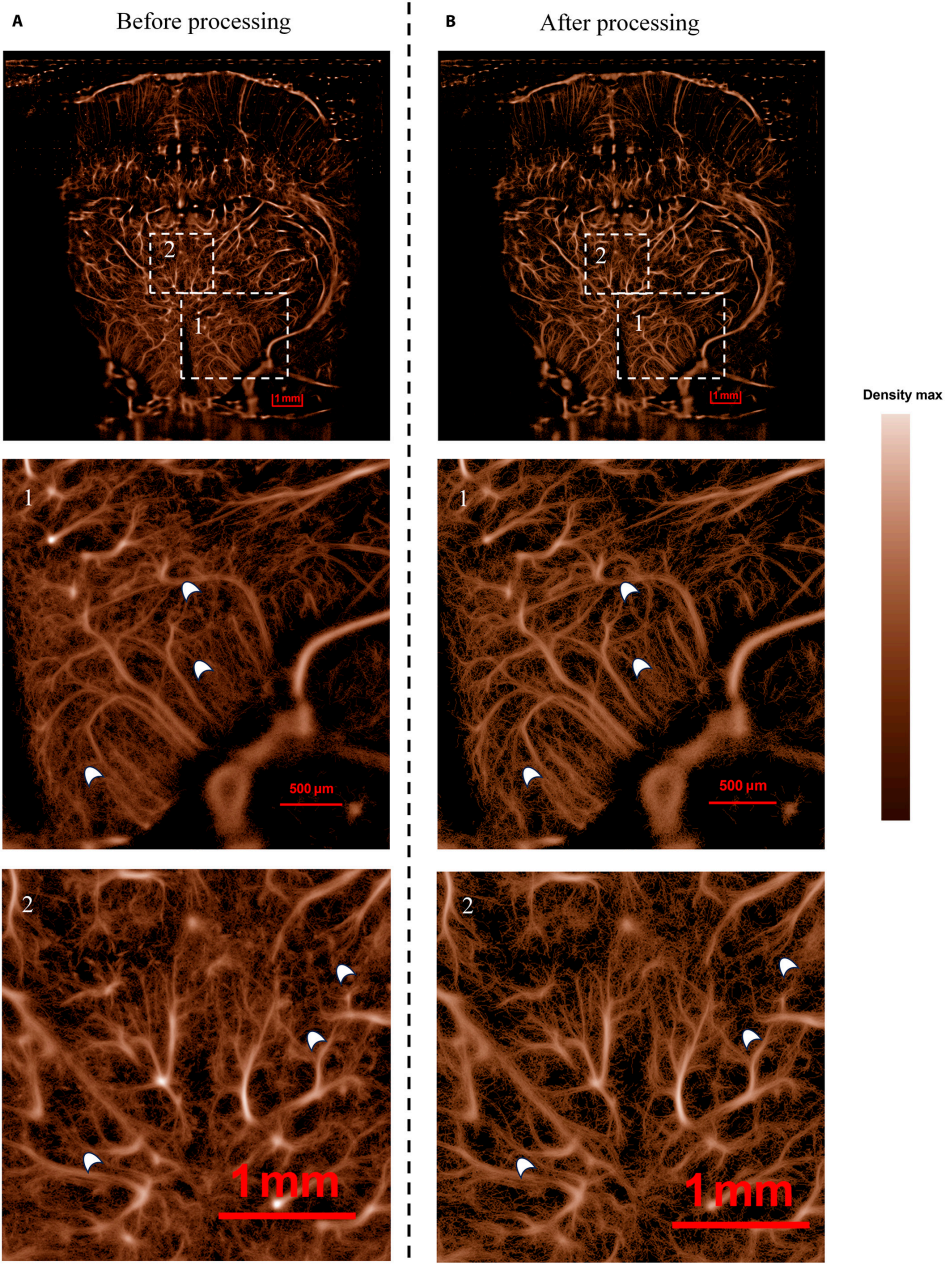
Comparison of microvascular density images of the rat brain (A) before processing and (B) after processing.

**Fig. 4.**
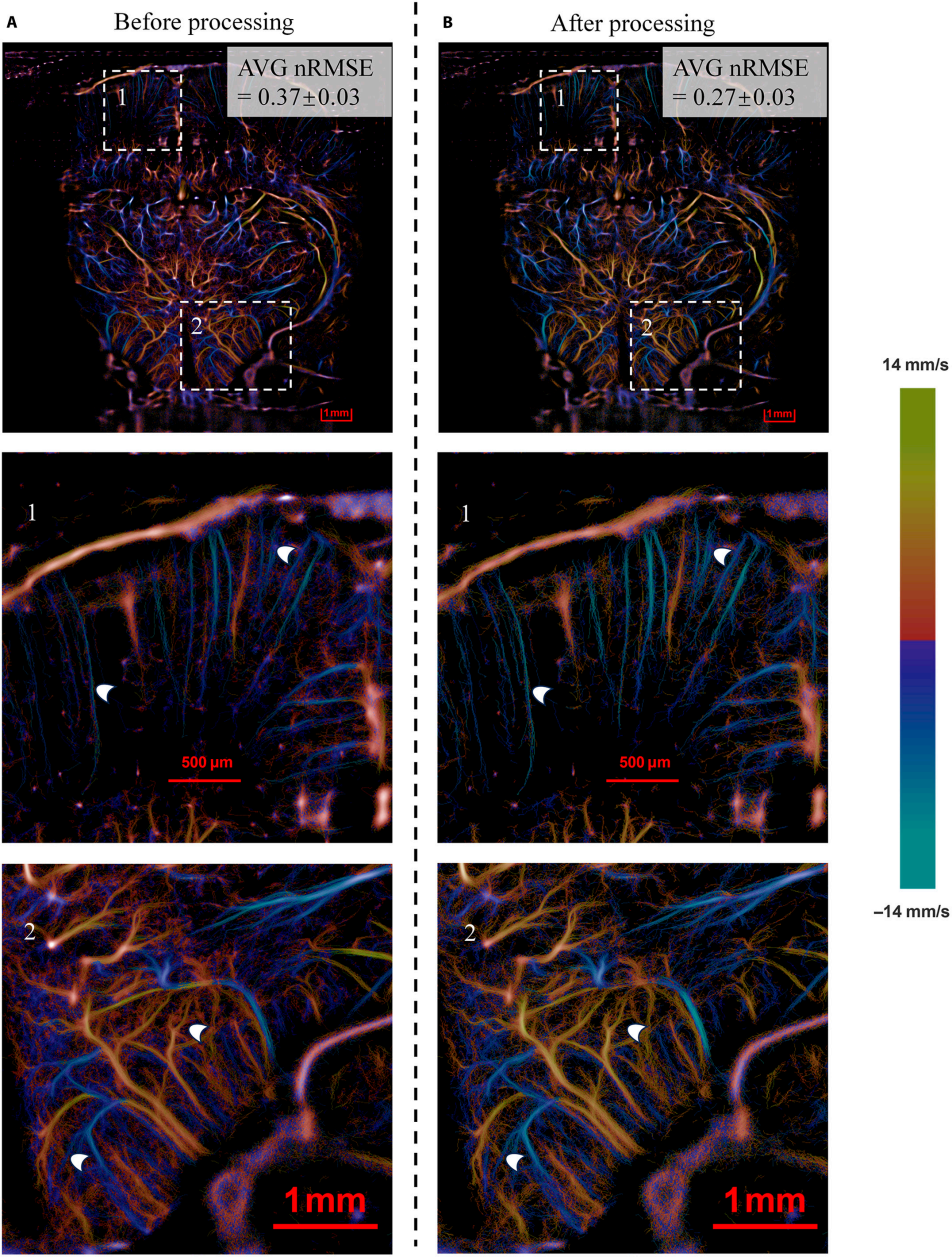
Comparison of microvascular flow velocity images of the rat brain (A) before processing and (B) after processing.

Figures 3 and 4 present a comparative analysis of the density and velocity maps in rat brain imaging obtained through the ULM algorithm, comparing results with and without vc-Kalman prediction processing. The enhanced vc-Kalman method, which integrates multiple parameters of microbubble characteristics including their positional statistical predictions and brightness features, demonstrates significant improvements in both the reliability and precision of microbubble localization and tracking performance.

It is obviously observed in Fig. 3, with respect to vascular morphology in the density map, that the proposed vc-Kalman algorithm produces a vessel distribution map with more distinct boundaries between different fine vessels. This improvement is closely related to the enhanced precision of predicting microbubble trajectories, which thus facilitates the reconstruction of high-quality ULM microvascular imaging.

Furthermore, the results shown in Fig. 4 also demonstrate that, compared to the traditional ULM method, the blood flow trajectory velocities estimated from the improved algorithm with vc-Kalman predictions exhibit superior tracking accuracy. In the figures, warm colors (e.g., yellow and red) indicate the upward blood flow direction, while cool colors (e.g., blue and green) represent the opposite flow direction, and the intensity changes of yellow and blue correspond to the velocity variations in both directions. It is clearly observed in the sample velocity maps that the average flow speed estimated for the enhanced ULM image (Fig. 4B) is higher than that obtained for the regular ULM image (Fig. 4A). Furthermore, we recalculated the normalized root mean square error (nRMSE) value for each pixel in the ULM images reconstructed with different algorithms and then averaged the nRMSE across the entire image. The results demonstrate that, after applying the vc-Kalman predictions, the overall mean nRMSE can decrease from 0.37 (using the traditional method, Fig. 4A) to 0.27 (Fig. 4B), corresponding to a 27% reduction. This indicates that the velocity constraint evaluation and adjustment may act as essential aspects in the accurate determination and refinement of microbubble trajectories, as it helps to filter out spurious or inaccurate paths that may result from measurement errors, noise, or other factors that could lead to inconsistent velocity patterns. Consequently, the dispersion of microbubble trajectory velocity estimations obtained by the vc-Kalman method can be significantly reduced, abnormal velocities (e.g., caused by mismatching) will be effectively suppressed, and the accuracy of microbubble tracking and velocity estimation will be significantly improved.

### Improved motion compensation in rat kidney

Although the application of the vc-Kalman method can accurately depict the microvascular structure and flow velocity distributions of fine vessels in rat brain (Figs. 3 and 4), it should be noticed that the blood vessels in brain are relatively static, while the greater challenge should still be faced by ULM in abdominal organs (e.g., liver and kidney), where nonnegligible signal deviation might be caused by significant physiological movements and deformations.

**Fig. 5.**
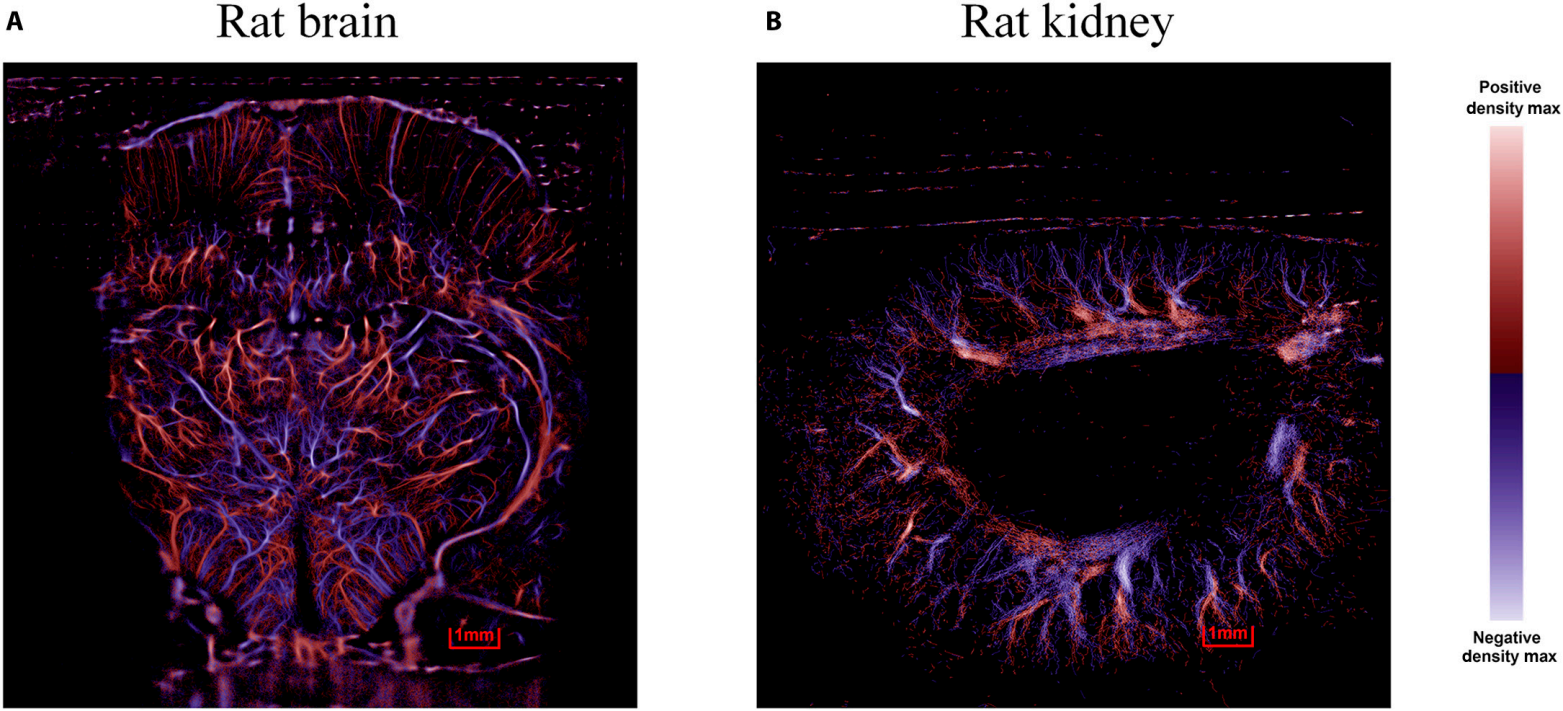
Microvascular density and direction map utilizing vc-Kalman filter prediction without motion compensation: (A) rat brain; (B) rat kidney.

In order to verify the improved motion compensation effect of the proposed method, sample ULM fusion images of vascular density and blood flow direction were generated for both rat’s brain and kidney, by using the vc-Kalman method without the motion compensation process. It is evident that the microvasculature in the kidney image (Fig. 5B) appears more chaotic and blurred than the brain (Fig. 5A), which are unavoidable outcomes resulting from more significant motion displacement experienced by rat kidneys. The comparison result suggests motion compensation is crucial for accurately capturing and representing blood flow features during ULM imaging for moving organs.

**Fig. 6.**
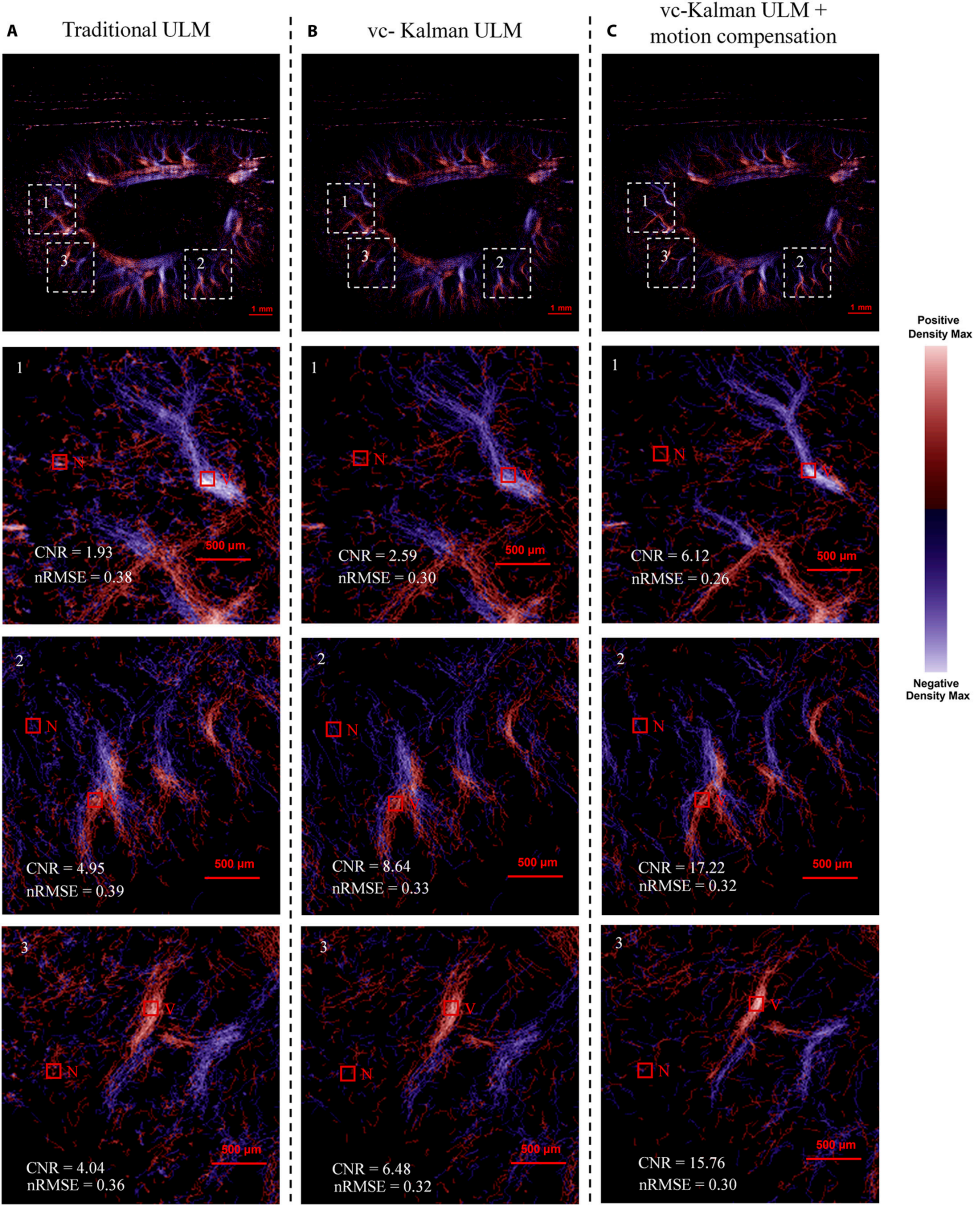
ULM results of the rat kidney processed by different methods: (A) traditional method; (B) vc-Kalman; (C) vc-Kalman method with dynamic programming-based cross-correlation motion compensation. Three typical observation regions (regions 1, 2, and 3 within white dashed boxes) are randomly selected to quantitatively evaluate CNR and nRMSE values obtained by different algorithms.

Therefore, a dynamic programming-based cross-correlation search was further applied in the current work to further improve the vc-Kalman algorithm, by extracting and discarding erroneous traces of blood flow trajectories. By quantitatively analyzing the changes in contrast-to-noise ratio (CNR) and nRMSE of ULM images obtained by 3 different algorithms, the comparison results confirm that the dynamic programming-based cross-correlation search process can achieve significant motion compensation (Fig. 6C), thereby successfully suppressing the noise and perturbations resulting from regular breathing and heartbeat as well as slight tissue movements in the kidney. As shown by the results, it can create clearer microvascular morphology and restore vascular continuity with higher velocity estimation accuracy, which exhibits performance superior to both traditional methods (Fig. 6A) and vc-Kalman alone (Fig. 6B). Specifically, we randomly selected 3 observation areas (regions 1, 2, and 3) in ULM images of rat kidney and compared the CNR and nRMSE values obtained by different algorithms. It can be clearly observed that compared to traditional algorithms (Fig. 6A), the application of vc-Kalman prediction can improve microvascular reconstruction quality and velocity estimation accuracy at a certain level (Fig. 6B and C), while the vc-Kalman algorithm combined with motion compensation (Fig. 6C) can further increase the CNR in traditional ULM images by about 2 to 3 times, while reducing nRMSE by about 20% to 30%. This indicates that the subpixel motion compensation algorithm can realize effective correction of tissue displacement in rat’s kidney while maintaining high computational efficiency (100,000 frames of data processing time is only about 1/100 of traditional methods).

## Discussion

The above results indicated that, by comprehensively integrating multidimensional feature information (e.g., brightness information, position prediction, and VD constraints), and combining dynamic programming-based cross-correlation method to improve the traditional ULM algorithm, the proposed vc-Kalman approach could be used to significantly enhance the accuracy of microbubble recognition, matching, and tracking, and address the signal deviation problem caused by physiological movements, which thereby effectively improved ULM imaging quality and enhanced the accuracy of blood flow analysis. In the following section, in-depth discussions would be conducted regarding various impact factors that might play an important role in optimizing the stability and accuracy of the current algorithm.

### Impacts of brightness information and Kalman filtering position prediction on ULM imaging quality

**Fig. 7.**
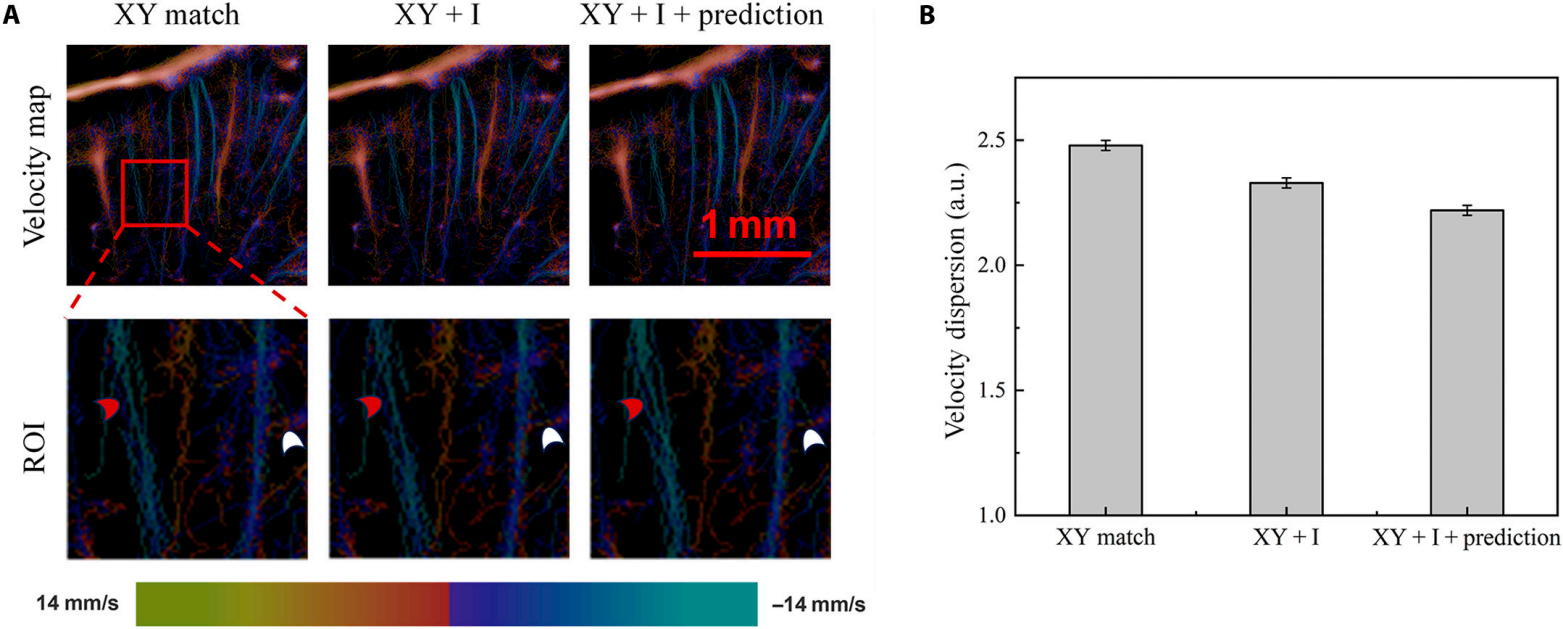
The impacts of brightness information and Kalman filter prediction on ULM results of rat brain: (A) comparison of velocity maps; (B) comparison of velocity dispersion.

By comparing with the ULM imaging results obtained by the traditional method, the results illustrated in Fig. 7 verify the enhancement effect of the improved algorithm on microbubble trajectory matching, which comprehensively takes into account both the bubble brightness and Kalman filtering position prediction information. In Fig. 7A, a capillary-rich area in the rat brain is used as the research object, and the blood flow velocity map and an enlarged view of the region of interest are shown from top to bottom, respectively. The columns from left to right present the results obtained by using the traditional ULM method, introducing microbubble brightness information during the matching process, and utilizing the enhanced algorithm by considering both brightness and Kalman filtering prediction information.

Comparing the results of the first 2 columns, it can be clearly observed that after introducing microbubble brightness information during the matching process, the vascular morphology indicated by the white arrow on the right in Fig. 7A is more accurately constructed with clearer boundaries. This improvement should be attributed to the addition of brightness information, which effectively suppresses noise interference during the imaging process to a certain extent. However, it should also be noted that when considering brightness information, some subtle vascular details related to relatively weak signal intensity may be affected and lost. Therefore, it is necessary to further utilize the Kalman filtering combined with bubbles’ historical information for position prediction to refine and correct the results of trajectory tracking, so that even weak signals of subtle vessels can be effectively supplemented, and high-precision visualization effects can be achieved (as shown by the red arrow on the left side of the figure).

Figure 7B further compares the velocity dispersion (VD) values calculated using 3 different matching methods. As defined in Materials and Methods, a larger VD value indicates the presence of more trajectory artifacts generated by incorrect matching, indicating poor ULM imaging quality and accuracy. It is clearly demonstrated that, as multidimensional features incorporating both microbubble brightness and Kalman filtering position prediction information are further considered, the trajectory inconsistency level can be significantly reduced. This indicates that the improved algorithm can effectively reduce the probability of incorrect matching of microbubble trajectories, significantly reduce the influence of image noise signals, and enhance the imaging quality and spatial resolution of ULM.

### Impact of VD constraint on the discriminability of the ULM vascular region

**Fig. 8.**
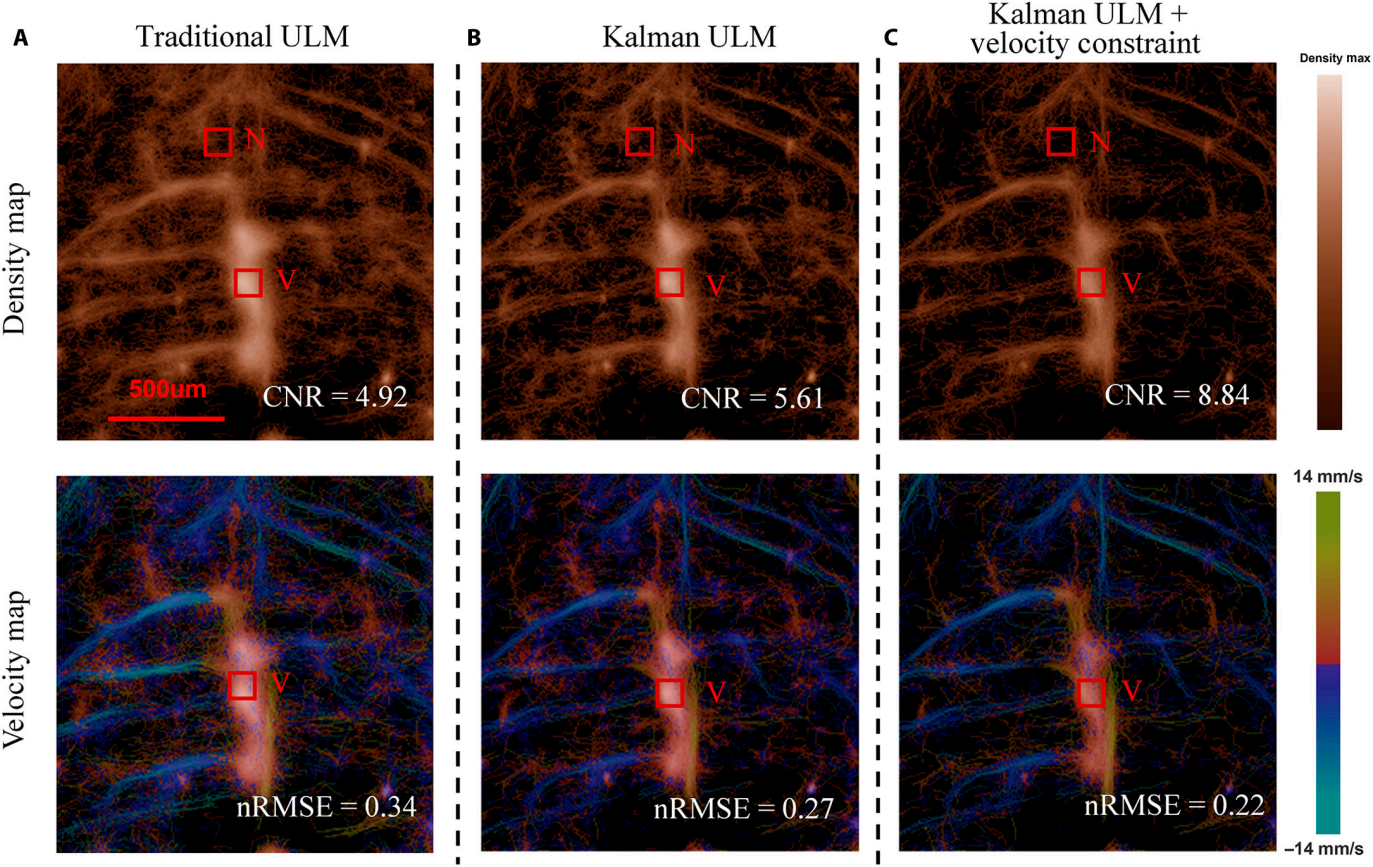
The comparison results between ULM results obtained with (A) traditional Kalman, (B) vc-Kalman, and (C) vc-Kalman with velocity constraints. “V” and “N” represent the selected vessel and noise region, respectively. The nRMSE is calculated for the whole image.

Figure 8 selects a capillary-rich region in the rat brain as a typical example and compares the vascular density and velocity mapping results obtained with the traditional ULM algorithm, the Kalman-filtering-based ULM method, and the Kalman-filtering-based ULM method with VD constraint processing, by quantitatively assessing the corresponding CNR and nRMSE values. According to the definitions of CNR and nRMSE in Materials and Methods, a higher CNR value indicates better differentiation between the vessel and noise regions, suggesting effective enhancement in the detectability of vascular structures within the image [16,24], and a lower nRMSE reflects less relative error in velocity estimation of microbubble trajectories and greater robustness of microbubble tracking efficacy. Figure 8A clearly exhibits that, for the rat brain image obtained with the traditional ULM method, there are an excessive number of cluttered capillaries with relatively low CNR (CNR = 4.92) and a high nRMSE level of 0.34. This phenomenon may be attributed to the presence of noise signals or displacement artifacts caused by physiological movements, which could result in many erroneous trajectories that do not conform to expected physical constraints. Then, if the Kalman method was applied without the application of VD constraint, the CNR of ULM image can be slightly enhanced to 5.61 with nRMSE decreased to 0.27 (Fig. 8B). In contrast, by applying the Kalman method with the VD constraint, the CNR of ULM image can be significantly enhanced to 8.84 (Fig. 8C), while nRMSE is greatly reduced to 0.22. These comparison results indicate that, the proposed VD constraint operation not only limits the magnitude of microbubble motion velocity, but also ensures that the velocity variations of microbubbles during 2 consecutive imaging frames fall within a physiologically reasonable range. More importantly, this method can effectively eliminate the possibility of erroneous matching with similar speeds but inconsistent directions, thereby further improving the accuracy and robustness of bubble localization and trajectory tracking. In other words, by further integrating a more effective velocity constraint process into the current multidimensional featured Kalman filtering algorithm, the microvascular structure and blood flow dynamics in rat brain can be more reliably and accurately characterized, providing solid data support for various biomedical studies.

### Improved suppression effect of the vc-Kalman algorithm on the frame rate dependence of blood flow analysis

Previous studies have demonstrated that a reduction in the frame rate of image acquisition is associated with a decline in the quality of ULM imaging, and can easily lead to the loss of microvascular morphology and underestimation of blood flow velocity [22]. This is because traditional ULM algorithms rely on the superposition and accumulation of extensive spatiotemporal information. Under low frame rate conditions, the spatiotemporal sampling rate is too low to provide sufficient historical displacement information for microbubble localization and trajectory tracking, thereby affecting the matching accuracy and reducing the accuracy of velocity estimation.

In contrast to traditional ULM methods, the current enhanced algorithm offers several substantial advantages. Firstly, the optimized multidimensional featured Kalman filtering algorithm can utilize brightness information to enhance the contrast between microbubbles and surrounding tissues. Meanwhile, by adopting extended Kalman filtering prediction, the future positions of microbubbles are estimated based on historical data, which not only provides additional dimensions for microbubble detection but also optimizes the trajectory tracking efficacy. As shown by the results, we can more accurately identify microbubbles’ position even at low frame rates, effectively improving the reliability of microbubble matching detection. Secondly, in the process of vc-Kalman filtering algorithm, the introduction of velocity constraints limits the reasonable range of motion velocity changes, eliminates the interference of abnormal trajectories caused by perturbation signals, and effectively reduces tracking errors associated with frame rate reduction. Moreover, in the process of reconstructing microbubble trajectories within moving organs, traditional methods are susceptible to excessive background noise at low frame rates, making it difficult to reconstruct complete bubble trajectories, resulting in a significant reduction in velocity evaluation accuracy. In contrast, our algorithm employs a dynamic programming-based cross-correlation method to better utilize limited sampling information and significantly enhance noise suppression efficiency, which thereby provides more accurate blood flow velocity information based on optimized trajectory reconstruction precision.

**Fig. 9.**
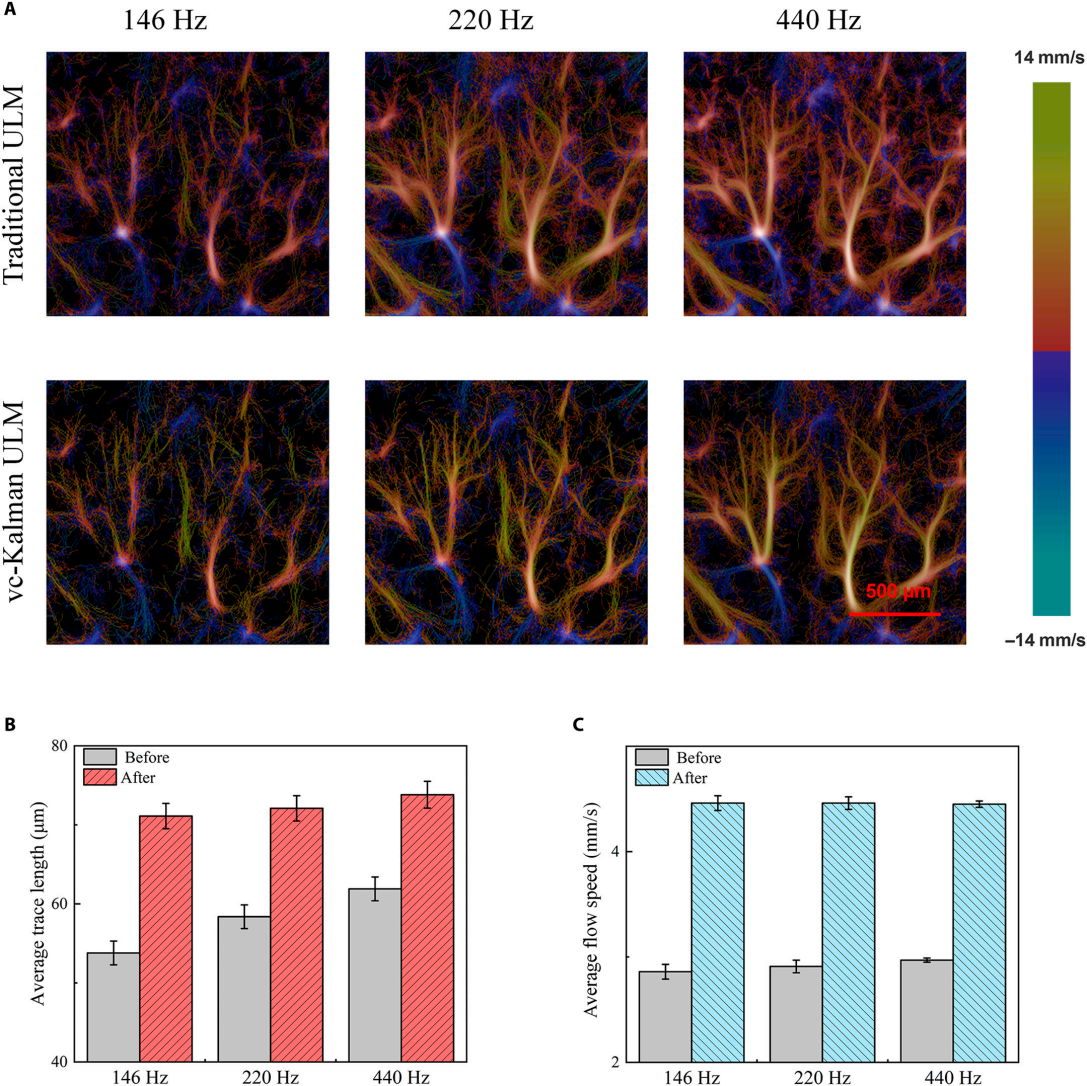
ULM results of rat brain based on Kalman filter prediction under different frame rate conditions: (A) comparison of rat brain velocity maps; (B) comparison of average trace length; (C) comparison of average flow speed.

In order to further verify the suppression effect of the vc-Kalman algorithm on the frame rate dependence in ULM imaging and blood flow analysis, systematical evaluations of ULM velocity maps and trajectory tracking effects obtained using traditional methods and the current vc-Kalman algorithm were performed for rat cerebral blood vessels, under varied frame rates. As shown in Fig. 9A, when the frame rate is reduced from 440 to 146 Hz, the imaging quality of the traditional method will significantly decrease, while the improved vc-Kalman algorithm can maintain relatively clear details of fine blood vessels even under low frame rate conditions. Figure 9B and C further compare the disparities in average trajectory length and average trajectory speed before and after applying vc-Kalman filtering at 3 different frame rates. With respect to the average trace length, at the lowest frame rate of 146 Hz, the improved vc-Kalman algorithm achieved an average trace length that is only 3.62% shorter than the result obtained at a 440-Hz frame rate, while the traditional method gave a significant reduction by 13.10%. Similarly, for the evaluation of average blood flow speed, when the frame rate was reduced to 146 Hz, the improved vc-Kalman algorithm predicted an average value that only changed by 0.22% compared to the result at 440 Hz, while the traditional method exhibited a significant decrease of 3.70%. These findings indicated that the imaging results of the vc-Kalman algorithm exhibited greater reliability at low frame rates, which effectively mitigated the incorrect identification or rupture of fine vascular ends, thereby enhancing the robustness of ULM trajectory reconstruction and the accuracy of blood flow analysis.

### Limitations and perspectives of the proposed method

In summary, the proposed algorithm represents a significant advancement in ULM technology. By quantitatively comparing the CNR values of ULM images obtained with the traditional Kalman filtering algorithm, it shows that, by comprehensively considering multidimensional features and implementing innovative motion compensation strategies, the current method can effectively overcome the signal bias problems caused by physiological motion and frame rate reduction. More importantly, based on the architecture improvement of graphics processing unit (GPU)-based commercial B-mode imaging platform and algorithm optimization based on C++ programming, we can significantly reduce the computation time of vc-Kalman ULM imaging jointed with motion compensation.

It should be noticed there are still some limitations to the work presented in this article. For example, the algorithm proposed here can only be applicable to organs with small physiological motion amplitudes (e.g., cerebral microvessels) or tissue displacement mainly affected by respiration (e.g., kidneys, liver, etc.). At this point, the motion compensation optimization matching algorithm based on dynamic programming can effectively correct the millimeter-level motion offset caused by deep breathing displacement within the B-mode imaging section. However, for organs that move rapidly and significantly (e.g., heart), it is still hard for this algorithm to obtain high-quality ULM images within an entire cardiac cycle. More detailed discussions regarding the motion artifact suppression capability and applicable motion range of the proposed method is provided in the Supplementary Materials. Moreover, the current work only demonstrates the improvement of the vc-Kalman method over traditional ULM algorithms on specific datasets, but does not quantitatively compare it with other tracking methods besides traditional Kalman filtering, such as particle filtering, deep learning, etc. In future work, we will further optimize the GPU acceleration strategy with more advanced parallel computing architecture and explore more improvement methods to enhance the imaging performance of the ULM algorithm. Consequently, it can have greater potential in practical applications, especially in extending to 3D microvascular blood flow imaging with even higher frame rates (e.g., 1,000 fps), which is expected to further improve the robustness and accuracy of ULM imaging and open new avenues for in-depth biomedical research and clinical applications.

## Materials and Methods

### Animal preparation

Adult male Sprague-Dawley rats, weighing 300 to 350 g (Beijing Vital River Laboratory Animal Technology, China), were utilized in the present work. These animals were housed under standard conditions (room temperature at 24 ± 2 °C, 60% ± 5% relative humidity, 12-h light–dark cycle) with sufficient food and water. All experimental procedures were conducted by certified technicians following the protocols approved by the Animal Ethics and Welfare Committee of Nanjing University.

During the experiments, the animals were anesthetized with isoflurane gas (3% initial concentration for induction, and 0.5% for maintenance). Two types of ULM imaging experiments were conducted, targeting relatively static rat brains and rat kidneys that are significantly affected by physiological movements, respectively. During the brain imaging, to improve the imaging quality by minimizing skull interference, a craniotomy was performed to remove the skull above the target imaging area of the rat’s brain, creating a 0.5 cm × 1 cm cranial window while protecting the dura mater to prevent brain inflammation. For kidney imaging, the fur surrounding the kidney region on the body surface of the anesthetized rat was removed with depilatory cream, so that potential scattering and absorption of ultrasound waves could be minimized as much as possible.

### Experimental designs

Figure 1 shows a sample picture of the 3D scanning system for animal experiments. The *x*-direction movement can be electronically controlled by the MATLAB (R2019a, MathWorks, USA) program for precise positioning, while the *y*- and *z*-direction movements are adjusted manually. Meanwhile, the probe holder enables flexible rotation for optimal angling adjustment. A heating pad was used to maintain the rat’s body temperature at 37 °C throughout the experiments. Near the head restraint, a tube was used to deliver anesthetic gas to the rat’s nose and mouth, while a side tube was used to ensure normal breathing.

According to the manufacturer’s instructions, the SonoVue (Bracco Diagnostics Inc, Italy) microbubble solution was prepared by injecting 5 ml of saline into a vial containing lyophilized powder and gently mixing it. During the experiments, 400 μl of ultrasound contrast agent (UCA) microbubbles was administered into the rat’s jugular vein via an intravenous catheter, with a constant rate of 80 μl/min for a duration of 5 min.

The imaging system mainly consisted of an ultrasound probe (L22-14L12N-6, Zhuhai Ecare, China) with 128 elements, a 0.1-mm pitch, and an 18-MHz center frequency, connected to an EC60 imaging module (Zhuhai Ecare, China). The imaging depth was set to 1.5 cm, and the final imaging area was approximately 1 cm × 1.5 cm. The pulse length for ULM imaging was set to 1.5 cycles with a system sampling rate of 50 MHz. Raw data acquisitions were repeated 5 times at each imaging site.

For the brain ULM experiments, to prevent near-field interference, the probe was positioned about 0.5 cm above the dura mater after the craniotomy procedure, with ultrasound gel (TM-100, Tianjin Jinya Technology Development, China) used as a coupling medium. Then, according to real-time B-mode observation, fine adjustment of probe position was performed to ensure that the entire brain was included in the image frame, with the midline of the coronal plane centered in the image.

For the kidney ULM studies, the rat was initially anesthetized with the fur around the kidney removed before the experiments, and ultrasound gel was then uniformly applied as a coupling medium. During the experiments, the ultrasound probe was precisely settled on the kidney region using this 3D positioning system, which provided highly accurate positioning and orientation of the probe to enable optimal imaging of the target area. Thereafter, the position and angle of the probe were finely adjusted according to real-time B-mode observations to ensure the entire kidney, including its complex vascular systems, was fully included in the image frame.

### System architecture improvement of the current imaging module

The traditional ULM algorithm is normally processed based on the MATLAB software running on specially designed research platforms (viz., Verasonics Vantage), which has bottleneck limitations such as a high computational cost and a long computation time. This study comprehensively optimized a commercial B-mode imaging platform to satisfy the practical demands for the acquisition and computation of large-scale ULM data (e.g., 100,000 frames of B-mode imaging RF data acquired in 217 s, 48 GB) with an ultrahigh frame rate (e.g., 440 fps). Firstly, breaking through the limitations of the commonly used Vantage platform and MATLAB software, C++ programming and OpenCL (Open Computing Language) was running on the Intel i7-13700KF and GPU (Nvidia GTX 1050) hardware platforms to significantly improve computational throughput, which, in turn, effectively accelerated the ultrasound beamforming and motion-compensated vc-Kalman ULM imaging processes. Meanwhile, the OpenMP (Open Multi Processing) multithreaded framework was adopted to achieve real-time parallel computing of 8 computing units, and the Intel Math Kernel Library was called to accelerate SVD operations, greatly improving the computing efficiency.

### Velocity-constrained Kalman filtering algorithm for enhanced bubble tracking

#### Subpixel-level bubble localization via the cosine function fitting method

The flowchart of vc-Kalman filtering for enhanced bubble tracking in motion-compensated ULM is illustrated in Fig. 2. In the initial bubble localization procedure, since the diameters of SonoVue microbubbles are typically less than 10 μm, individual microbubbles can only occupy a few pixels even in high-resolution images. Therefore, a cosine function fitting method was applied to address this spatial resolution challenge and enhance geometric center localization accuracy [24]. The geometric center coordinates were determined by identifying the maximum value of the fitted cosine function. The coordinate offset, represented by the parameter ξ = −𝛽/𝛼, and the intermediate parameters 𝛼 and 𝛽 were derived from the brightness intensity profiles of 3 adjacent pixels:

$$\alpha =\text{arccos}\left[\frac{I\left(-1\right)+I\left(1\right)}{2I\left(0\right)}\right],$$ (1)

$$\beta =\text{arccos}\left[\frac{I\left(-1\right)-I\left(1\right)}{2I\left(0\right)\text{sin}\alpha }\right].$$ (2)

where I(0) is the brightness of the pixel with the maximum intensity in the region, and I(−1) and I(1) are the brightness values of the 2 neighboring pixels in the *x* or *y* direction. Compared with traditional interpolation techniques, this cosine function fitting method can improve computational speed and enhance the real-time performance of ULM applications. Meanwhile, by comprehensively considering the pixel brightness information, the geometric center of microbubbles can be more accurately determined at the subpixel level, which helps to achieve high-precision super-resolution imaging.

### Enhanced bubble tracking via extended Kalman filter integrates multidimensional features

In traditional ULM algorithm, the microbubble tracking and velocity mapping processes rely only on the statistical information derived from bubbles’ historical observation positions (as shown in step 4 of Fig. 2). However, this simple trajectory matching method could easily lead to misidentifying different microbubbles as the same, thereby resulting in incorrect blood flow imaging. To address this challenge, a more extended microbubble tracking method based on Kalman filter [25,26] has been proposed. This method integrates multidimensional features of bubbles, such as the bubbles brightness information as well as their statistical observation information, to achieve more robust and accurate bubble tracking. Only for newly appearing microbubbles, their future position prediction still relies on the statistical information derived from the observed data. For most microbubbles that have existed for some time, the extended Kalman filtering method can more accurately predict their future positions based on the bubbles’ historical localization and velocity data. Compared with the traditional method, the extended Kalman filtering approach enables bubble matching within the possible range of their motion, rather than simply matching the nearest one. This strategy greatly reduces the matching errors that may occur when bubbles move too fast, or their concentration is too high (as illustrated by the matching diagram in the blue box in Fig. 2). To be more specific, a wrong match usually only considers the proximity of microbubble positions for matching. However, the effective state of a microbubble is actually determined by a weighted combination of its position and brightness. By considering brightness, the proposed algorithm can more accurately reconstruct complex microbubble trajectories, thereby achieving high-quality ULM blood flow imaging and analysis.

The multidimensional featured Kalman filter is introduced as Eq. 3. The motion state of the microbubble at time 𝑘 can be predicted from its state at the previous time point (𝑘−1):

Sk=xkykIkdxkdyk=1001001001001000001000001xk−1yk−1Ik−1dxk−1dyk−1+wxk−1wyk−1wIk−1wdxk−1wdyk−1, (3)

where 𝑥(𝑘) and 𝑦(𝑘) represent the positional coordinates of the microbubble at time instant 𝑘 in the *x* and *y* directions, respectively, while d𝑥(𝑘), d𝑦(𝑘), and I(𝑘)denote the corresponding velocity and brightness values, along with system noises affecting these parameters represented by the variables $w_{x}$, $w_{y}$, $w_{I}$, $w_{dx}$, and $w_{dy}$. Notably, the unit for displacement is in pixels, and the unit for velocity is pixels per frame interval. This motion state includes not only positional information but also brightness and velocity.

The updated predicted value can be expressed as:

$${\hat{S}}_{k,k}={\hat{S}}_{k,k-1}+K_{k}\left(z_{k}-{\hat{S}}_{k,k-1}\right),$$ (4)

$$P_{k,k}=\left(z_{k}-{\hat{S}}_{k,k-1}-K_{k}H_{k}\right)P_{k,k-1}{\left(z_{k}-{\hat{S}}_{k,k-1}-K_{k}H_{k}\right)}^{T}$$ (5)

Here, the superscript denotes the predicted values. ${\hat{S}}_{k,k}$ is the estimated state at time step *k*, and ${\hat{S}}_{k,k-1}$ is the predicted state at time step *k−*1. $P_{k,k}$ is the covariance matrix of the current state estimation, and $P_{k,k-1}$ is the prior estimate covariance matrix of the current state (predicted at the previous state)**.** Specifically, **K**_**k**_, **z**_**k**_, and **H**_**k**_ represent the Kalman gain matrix, measurement matrix, and observation matrix, respectively. The Kalman Gain matrix is given by:

$$K_{k}=P_{k,k-1}\cdot H_{k}^{T}\cdot {\left(H_{k}P_{k,k-1}H_{k}^{T}\right)}^{-1},$$ (6)

where the superscript “T” represents the transpose of a matrix and the superscript “-1” represents the inverse of a matrix.

In other words, for each element in the matrix, in the case where the Kalman gain is equal to 0, the updated predicted value is solely determined by the preceding predicted state. Conversely, when the Kalman gain coefficient is 1, the updated predicted value is entirely dictated by the measurement. This principle elucidates the role and significance of the Kalman gain in the context of the Kalman filter algorithm, which is crucial for optimally combining the predicted and measurement information to obtain more accurate estimates. The covariance matrix plays a vital role in quantifying the uncertainty associated with the state estimate, and the observation matrix defines the relationship between the measurement matrix and the underlying state variables. The measurement matrix provides the external information that is incorporated into the estimation process, and the Kalman gain acts as a weighting factor that adaptively adjusts the contribution of the measurement matrix based on the relative uncertainties of the prediction and the measurement. Through this iterative process of prediction and update, the Kalman filter is able to track and estimate the dynamic state of a system with improved accuracy and reliability.

**Fig. 10.**
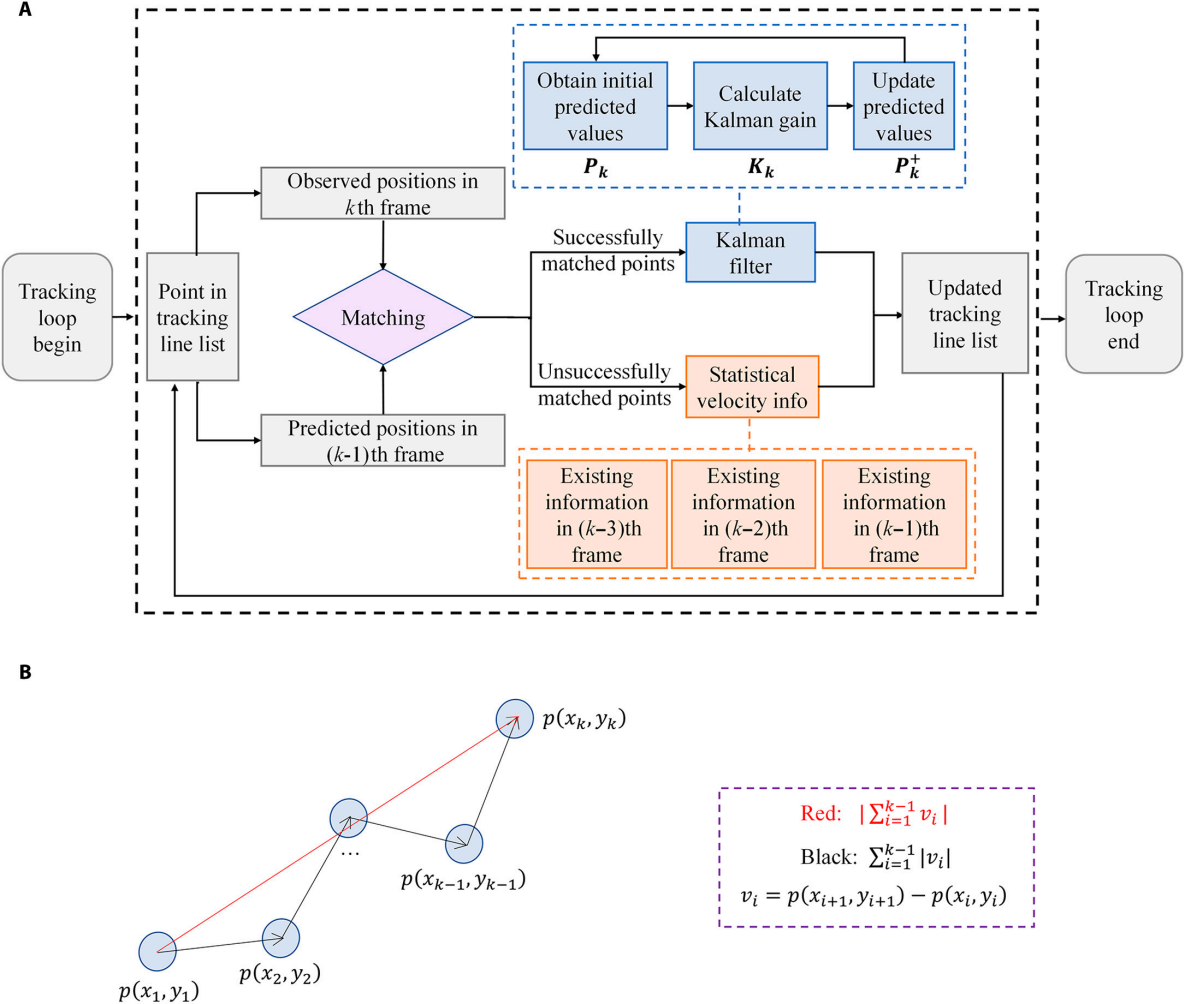
Critical procedures for the enhanced vc-Kalman approach. (A) Flowchart of the iterative process for tracking microbubble trajectories, combining both statistical observation information and multidimensional featured Kalman-filter-predicated positions; (B) schematic diagram of the velocity constraint analysis.

Figure 10A illustrates the innovative procedures for the precise tracking of microbubble trajectories using the extended Kalman filtering method. Firstly, based on the statistical data of subpixel microbubble observation positions, the initially predicted microbubble trajectories are acquired. Then, the tracking line list is constructed to initiate the correction and optimization processes of trajectory tracking. During the iterative process, a matching operation is performed between the predicted and actually measured positions of bubbles in the *k*th frame. In the case that the actually measured position of a bubble in the subsequent frame remained within the confines of its predicted trajectory, it is classified as a successful prediction. For such bubbles, their positions are further optimized and updated with the data predicted by the Kalman filter, by comprehensively taking account of multiple features including the historical velocity, brightness, and other relevant information from the preceding trajectories. The Kalman filter, operating on the principles of recursive estimation, utilizes the prior knowledge of the system’s dynamics and the observed data to generate more accurate estimation of thebubbles’ positions.

Conversely, if the actual measured position of a bubble deviates from the predicted trajectory (usually because it could be a newly appeared microbubble), it is classified as a failed prediction. In such cases, the predicated positions of these bubbles can only be updated based on their statistical velocity information in the past frames, thereby allowing the generation of new trajectories. Specifically, in order to avoid missing detection at individual frames and improve the reliability of overall microbubble position estimation, especially under non-Gaussian noise conditions, historical information from 3 preceding frames (viz., *k−*1, *k−*2, and *k−*3) are incorporated here to provide a smoother velocity estimate, compensate for occasional detection loss, and enhance prediction accuracy without sacrificing temporal resolution.

Eventually, the iterative process, which combines both the powerful Kalman filtering approach for pre-existing microbubbles and the statistical velocity information for new trajectory points, will be carried out frame by frame until all frames had been processed. Through this continuous cycle of prediction, comparison, and correction, the extended Kalman filtering method is able to adaptively adjust its estimates and improve the accuracy of microbubble trajectory tracking over time, accounting for the uncertainties in the bubble motion and measurement environment.

### Velocity-constraint evaluation and adjustment

Furthermore, the VD constraint can be conducted after evaluating the extent of distortion or irregularity in the path traversed by the microbubble, which is quantitatively analyzed according to the following VD coefficient defined as:

$$\mathrm{VD}=\frac{\sum_{i=1}^{k-1}|\;v_{i}\;|}{|\;\sum_{i=1}^{k-1}v_{i}\;|}\leq 2,\;v_{i}=p\left(x_{i+1},\;y_{i+1}\right)-p\left(x_{i},\;y_{i}\right),$$ (7)

where 𝑝(𝑥_𝑖_, 𝑦_𝑖_) represents the position of the *i*th microbubble.

In the regular case of microbubble movement within blood vessels, it is a well-established fact that blood vessels generally do not exhibit frequent and erratic twisting or contorting behaviors under normal physiological conditions. According to preliminary studies where synthetic noisy trajectories were simulated in the ULM calculations, we have found that, within a continuous sequence of *k* frames (*k* ≥ 3, *k*∈**N**), the sum of the magnitudes of microbubble velocities between adjacent frames (black line in Fig. 10B) should not deviate by more than 100% from the magnitude of the vector sum of these velocities (red line in Fig. 10B). If the aforementioned condition is not fulfilled, the corresponding trajectory is considered erroneous and, consequently, should be discarded. This velocity constraint evaluation and adjustment are an essential aspect in the accurate determination and refinement of microbubble trajectories, as it helps to filter out spurious or inaccurate paths that may result from measurement errors, noise, or other factors that could lead to inconsistent velocity patterns. By imposing this constraint, the analysis can focus on more reliable and physically meaningful trajectories, enhancing the overall quality and reliability of the microbubble tracking and analysis process.

The quality of reconstructed image can be quantitatively assessed by calculating the CNR [27] between 2 distinct regions: the vessel area (which primarily contains blood vessels) and the noise area (which is primarily composed of noise components). The formula for calculating CNR is:

$$\mathrm{CNR}=\frac{\left|E_{V}-E_{N}\right|}{\sigma_{N}},$$ (8)

where *E*_V_ and *E*_N_ represent the average intensities of the selected vessel and noise region, respectively, and 𝜎_𝑁_ denotes the standard deviation of the noise region. The CNR normalizes the contrast with respect to the noise level, allowing for a more meaningful comparison and evaluation of the effectiveness of the processing in enhancing the visibility and distinguishability of the vessel region from the background noise. In diagnostic applications, high CNR can highlight blood vessels from the surrounding tissue background, making it easier for uses to determine the shape, location, and presence of lesions in blood vessels.

In addition, another representative indicator parameter, the nRMSE, is also adopted to quantify the relative error in velocity estimation of microbubble trajectories and evaluate the accuracy and robustness of different ULM tracking algorithms. In the present work, each ULM reconstructed image was generated by accumulating microbubble trajectories from 100,000 consecutively acquired frames. Therefore, for each pixel within the vessel in the image, the nRMSE could be calculated as follows:

$$\text{nRMSE}=\frac{\sqrt{\frac{1}{N}\sum_{i=1}^{N}{\left(V_{i}-V_{\text{mean}}\right)}^{2}}}{\left|V_{\text{mean}}\right|},$$ (9)

where *N* represents the total number of sample points within the selected observation region, $V_{i}$ denotes the velocity vector obtained from a single tracking event, and $V_{\text{mean}}$ is the mean velocity vector calculated from multiple tracking results. By normalizing the root mean square error, this parameter can eliminate the influence of the absolute speed of blood flow on the evaluation and directly reflects the dispersion degree of the estimated velocities relative to the mean value, thereby representing the magnitude of trajectory tracking errors. Theoretically, if microbubble tracking accuracy is high enough, the true flow velocity at a single pixel within the vessel can be considered constant under steady-state conditions. Thus, a smaller nRMSE indicates higher consistency among velocity estimations, lower tracking errors, and greater algorithm robustness.

### Dynamic programming-based cross-correlation motion compensation

We have observed in practical applications that the motion compensation operation module is the most time-consuming in the ULM algorithm. If we refer to the traditional ULM algorithm based on MATLAB software programming on the Vantage platform, the processing time for 100,000 frames of RF data would cost as long as 6,337 s; even the MATLAB Parallel Computing Toolbox was adopted. More importantly, the motion compensation module would take up to 44.5% of the total computation time (viz., 2,819 s). Therefore, in addition to improving the hardware platform of the imaging module based on the GPU system, another key task of the current study was to optimize the algorithm for the motion compensation function to significantly shorten its computation time to meet the real-time requirements of practical applications.

To achieve this purpose, motion compensation was performed via a normalized cross-correlation search technique based on dynamic programming principles, which aimed to isolate and eliminate erroneous trajectories that may arise due to slight tissue movements. Specifically, the process entailed conducting matching searches on blocked B-mode images. The objective of these searches was to precisely ascertain the relative movement existing between the reference and target frames. To fulfill this task, the middle frame of the entire time series was selected as the reference frame. Subsequently, each subsequent frame would be compared to the reference frame, and then corresponding displacement images were generated to provide quantitative assessment of the displacement that occurred between the frames. For individual localized microbubbles in each frame, the motion compensation could be achieved by subtracting the corresponding displacement vector and eventually constructing position-aligned microbubble localization image.

In in vivo studies, the relative displacement of the entire image were usually not constant. Therefore, to accurately account for these displacements, 2 2-dimensional (2D) displacement images were utilized to depict the displacements occurring in the *x* and *y* directions, respectively. Typically, a normalized correlation coefficient [28] was used to characterize the similarity between the reference and the target frame regions:

$$\begin{array}{c} R_{\mathrm{nc}}\left(\delta x,\;\delta y\right)=\\ \frac{\iint \left[S_{r}\left(x,\;y\right)S_{d}\left(x+\delta x,\;y+\delta y\right)\right]dxdy}{\sqrt{\iint {\left[S_{r}\left(x,\;y\right)\right]}^{2}dxdy\iint {\left[S_{d}\left(x+\delta x,,,\;,y+\delta y\right)\right]}^{2}dxdy}}, \end{array}$$ (10)

where *S*_r_ and *S*_d_ represented the reference and target frame images, respectively, and (δ*x*, δ*y*) was the displacement vector. In other words, the process of determining the displacement image entailed iteratively traversing through each possible displacement vector to find the maximum value of *R*_nc_, which indicated the most likely or optimal displacement that maximized the correlation between the reference and target frames.

For microvascular flow applications, a high level of precision is required for the displacement vector, specifically to the subpixel level. To fulfill this purpose, *R*_nc_ was firstly calculated with pixel-level accuracy. Then, with the application of parabolic fitting, the interpolation process was performed to determine the displacement vector corresponding to the maximum of *R*_nc_ at the subpixel level within a 3×3 pixel range:

$$\xi =\frac{R_{\mathrm{nc}}\left(-1\right)-R_{\mathrm{nc}}\left(1\right)}{2\left[R_{\mathrm{nc}}\left(-1\right)-2R_{\mathrm{nc}}\left(0\right)+R_{\mathrm{nc}}\left(1\right)\right]}.$$ (11)

Here, 𝜉 represented the subpixel offset in the *x* or *y* direction. (0) was the maximum value of *R*_nc_ within the region, while (−1) and *R*(1) were the values of *R*_nc_ at the pixel level for the 2 neighboring points, respectively. By adopting this parabolic fitting approach and using the values at these neighboring points, we are able to estimate the subpixel offset with greater accuracy. This subpixel-level accuracy in determining the displacement vector is crucial for accurately characterizing the microvascular flow. It allows for a more detailed and precise understanding of the movement and behavior of microbubbles within the microvasculature, which is essential for applications such as quantitative analysis of blood flow, assessment of vascular function, and detection of microcirculatory abnormalities. Additionally, 2D prefix sum method [29] was adopted to reduce computational complexity. This method was designed to transform the double summation of the integral calculation into a series of addition and subtraction operations, greatly enhancing computational efficiency.

After the construction of a displacement vector image, an optimized cross-correlation search process was further conducted to rapidly explore all possible matching offset and identify the most likely displacement deviation of the object being tracked (in this case, microbubbles) caused by physiological movements, thereby minimizing the overall error between the frames and enabling more accurate and reliable analysis of the microbubble motion and trajectories. Moreover, since the displacement image exhibits a certain degree of continuity, a maximum normalized cross-correlation searching process was performed based on the combination of dynamic programming [30] and parabolic interpolation. For parabolic interpolation, both along the *x*-axis and *y*-axis, at least 3 sampling points would be required to accurately fit a parabola and precisely determine the central location within the searching range at subpixel resolution. Larger windows would increase computational cost without significantly improving localization accuracy. Therefore, rather than conducting a comprehensive search across the entire image space, a 3×3 window size was selected to offer the best balance between the computational efficiency and subpixel accuracy in practical implementation.

Eventually, after combining C++ program optimization, OpenMP multithreaded parallel computing, and OpenCL-based GPU acceleration, we successfully achieved motion compensation for 100,000 frames of data within just 21 s, which was 134 times more efficient than the traditional method (2,819 s), and the overall computation time of the vc-Kalman ULM algorithm with motion compensation was only 67 s, which was 94.6 times higher than the traditional one (6,337 s) and even much lower than the data acquisition time (217 s), making this method capable of quasi real-time processing and exhibiting good computational performance in ultrahigh frame rate ultrasonic imaging circumstances.

## Data Availability

All data could be acquired from J.T. upon request.
