# Supplementary material for: Velocity-Constraint Kalman Filtering for Enhanced Bubble Tracking in Motion-Compensated Ultrasound Localization Microscopy
Source: Research (Wash D C). 2025 Jun 4;8:0725. doi: 10.34133/research.0725 (PMC12136335; doi:10.34133/research.0725)
Supplement: Supplementary 1 — Supplementary Text Figs. S1 and S2 [file research.0725.f1.docx]

Supplementary Material

**Discussion on the applicability of vc-Kalman ULM algorithm with motion compensation**

In general, the key impact factor of the current vc-Kalman ULM algorithm with motion compensation lies in the imaging cross-sectional thickness of the ultrasound probe used practically. For the probe used in the current study, the imaging cross-sectional thickness of the ultrasound probe (L22-14L12N-6, Zhuhai Ecare, China) used here was measured to be approximately 1.8 mm according to the guidelines of GB10152-2009 "B-mode Ultrasonic Diagnostic Equipment" (see Fig. S1). Theoretically, as long as the motion trajectory of microbubbles is within the range of the ultrasound imaging section, its motion deviation can be able to be corrected via motion compensation algorithms. Therefore, the current experimental system should be able to achieve millimeter-level motion compensation.


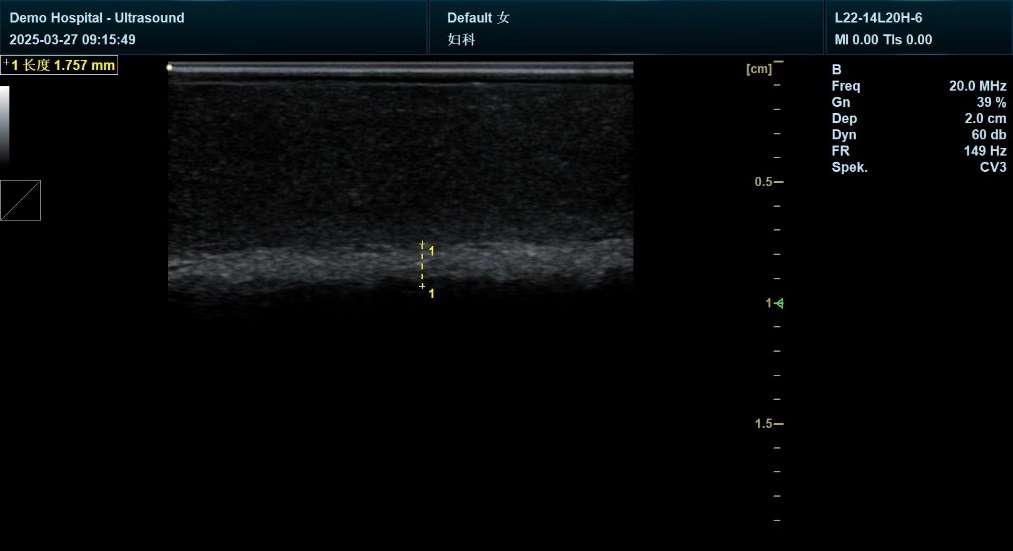


Fig. S1 the imaging cross-sectional thickness of the ultrasound probe

Moreover, the displacements in the rat kidney caused by respiration and heartbeat were both measured in the *x*-direction and *y*-direction, respectively, during a complete respiratory cycle. The experimental data shows in Fig. S2 demonstrate that, the respiratory cycle of rat kidney is about 3.6 s, and the cardiac cycle is about 0.18 s. It is obvious that the tissue displacement caused by respiration is much greater than that caused by heartbeat. Specifically, during the heartbeat cycle, the average displacement of the tissue is about 25 μm. Within one respiratory cycle, the deep breathing displacement accounts for about 11% of the entire period, while the remaining 89% of the time can be considered as the steady breathing phase. During the steady phase of respiration, the maximum displacement of mouse kidney tissue is about 100 μm, and the maximum tissue displacement caused by deep respiration can reach over 500 μm, but it is still less than the imaging cross-sectional thickness of ultrasound probe (1.8 mm). Therefore, the motion compensation algorithm proposed here can still effectively correct motion artifacts at this stage.


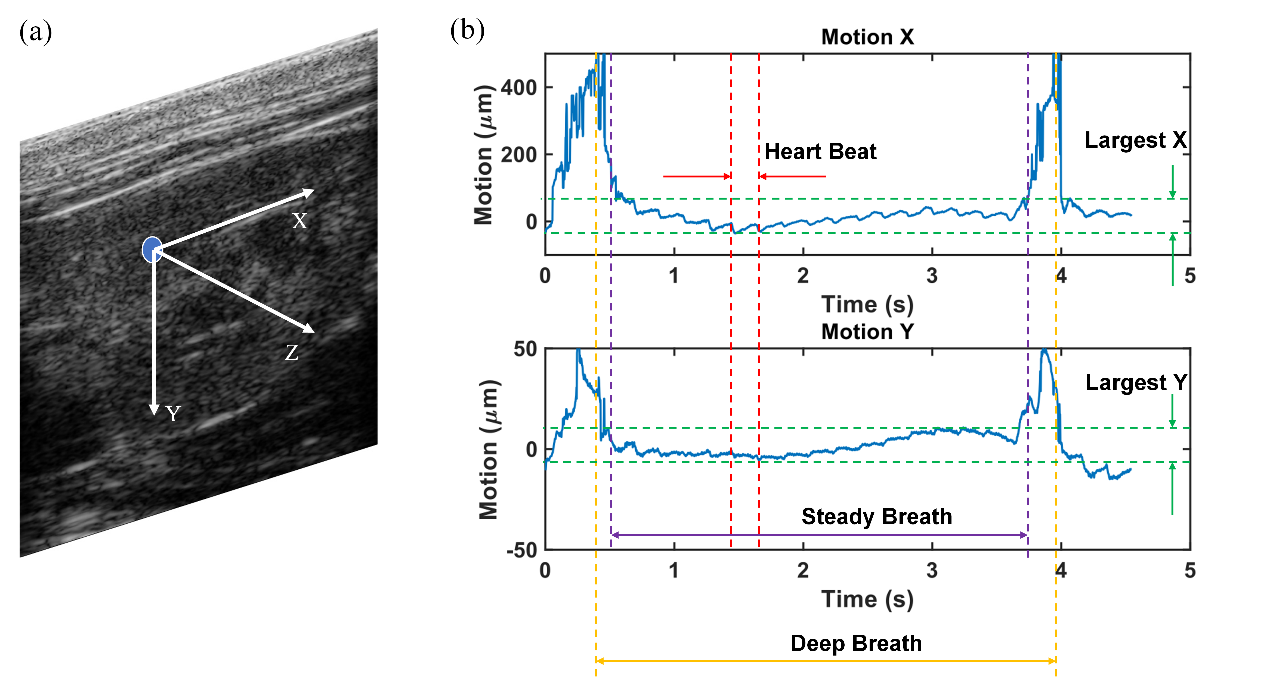


Fig. S2 The displacements in the rat kidney caused by respiration and heartbeat during a complete respiratory cycle. (a) A sample B-mode image; (b) The sample displacement curves extracted for a typical point (the blue dot in Fig. S2a) along the X and Y directions.
